# Improving Quality and Safety in Aged Care through Digital Innovation and Data-Driven Interventions

**DOI:** 10.1093/geroni/igaf122.4142

**Published:** 2025-12-31

**Authors:** Francois Xavier Duchateau, Aileen Waton, Mel White, Ana Isabel Fernandez Castello, Siobhan Mackintosh, Anne Lepetit

**Affiliations:** BUPA, London, England, United Kingdom; Bupa UK, London, England, United Kingdom; Bupa Australia, Melbourne, Victoria, Australia; Sanitas, Madrid, Castilla y Leon, Spain; Bupa, London, England, United Kingdom; Bupa, London, England, United Kingdom

## Abstract

Introduction Improving the quality of care while minimizing risks remains a core objective for healthcare organizations. Aged care (AC) presents unique challenges associated with the older population, particularly polypharmacy, nutritional deficits, and reduced mobility. This study illustrates how digitalization and data-driven approaches can support quality improvement in AC, through the evaluation of 3 recent initiatives. Methods We are a non-profit international healthcare provider that delivers AC. In the UK, an Electronic Medication Administration Record (eMAR) system was gradually implemented across 120 care homes (CH) between June 2023 and June 2024. The effectiveness of this intervention was assessed through reported medication error rates. In the Australia, 57 CH are supported by 13 Wellness Hubs staffed by 24/7 Nurse Practitioners, who coordinate multidisciplinary teams including dietitians, physiotherapists, wound care specialists... As a quality indicator, we measured the time to healing for pressure ulcers. In Spain, 45 CH adopted a digital care model including voice assistants (Alexa) in each room to provide real-time care instructions, based on individualized care plans. The incidence of falls per 1,000 bed-days was reported. All data were consolidated using Power BI dashboards. Results Following the implementation of eMAR, minor medication errors decreased by 7%, and moderate to major errors declined by 50%. In APAC, the healing time for pressure ulcers was reduced by 42 days. In Spain, the rate of falls decreased to below 5 per 1,000 bed-days. Conclusion These findings suggest that the implementation of tailored, technology-enabled initiatives can significantly enhance quality and safety in AC.

